# Recent trends in microbial flavour Compounds: A review on Chemistry, synthesis mechanism and their application in food

**DOI:** 10.1016/j.sjbs.2021.11.010

**Published:** 2021-11-12

**Authors:** Deepak Kumar Verma, Shayma Thyab Gddoa Al-Sahlany, Alaa Kareem Niamah, Mamta Thakur, Nihir Shah, Smita Singh, Deepika Baranwal, Ami R. Patel, Gemilang Lara Utama, Cristobal Noe Aguilar

**Affiliations:** a Agricultural and Food Engineering Department, Indian Institute of Technology Kharagpur, Kharagpur 721302, West Bengal, India; b Department of Food Science, College of Agriculture, University of Basrah, Basra City, Iraq; c Department of Food Technology, School of Sciences, ITM University, Gwalior 474001, Madhya Pradesh, India; d Division of Dairy Microbiology, Mansinhbhai Institute of Dairy & Food Technology-MIDFT, Dudhsagar Dairy Campus, Mehsana-384 002, Gujarat, India; e Department of Nutrition and Dietetics, University Institute of Applied Health Sciences, Chandigarh University, Chandigarh 140413, Punjab, India; f Department of Home Science, Arya Mahila PG College, Banaras Hindu University, Varanasi, Uttar Pradesh 221005, India; g Faculty of Agro-Industrial Technology, Universitas Padjadjaran, Sumedang 45363, Indonesia; h Center for Environment and Sustainability Science, Universitas Padjadjaran, Bandung 40132, Indonesia; i Bioprocesses and Bioproducts Group, Food Research Department, School of Chemistry. Autonomous University of Coahuila, Saltillo Campus, 25280 Coahuila, México

**Keywords:** Aroma compounds, Biotransformation, Microbiota, Acetaldehyde, Flavour characteristics

## Abstract

Aroma and flavour represent the key components of food that improves the organoleptic characteristics of food and enhances the acceptability of food to consumers. Commercial manufacturing of aromatic and flavouring compounds is from the industry's microbial source, but since time immemorial, its concept has been behind human practices. The interest in microbial flavour compounds has developed in the past several decades because of its sustainable way to supply natural additives for the food processing sector. There are also numerous health benefits from microbial bioprocess products, ranging from antibiotics to fermented functional foods. This review discusses recent developments and advancements in many microbial aromatic and flavouring compounds, their biosynthesis and production by diverse types of microorganisms, their use in the food industry, and a brief overview of their health benefits for customers.

## 1 Introduction

Flavours and aromas play a major role in our everyday lives. They are available in food and cosmetics. Nowadays, demand for natural ingredients rather than a chemical is increasing and it is the same for flavour compounds also (Roman et al., 2017). Flavouring compounds in the food, perfumes, and pharmaceutical industries are widely used. In general, plant compounds are the main sources of natural flavour though some of them are also synthesized chemically. Culturing plant cells is a promising process for flavour and aroma production. This method is based on the biochemical, genetic, and totipotential capabilities of plant cells (Ayseli and Ayseli, 2016, Zakaria and Kamal, 2016).

On the other side, biotechnology advances make it possible to synthesize natural flavours economically and successfully at a commercial scale. Enzymes are used for biotransformation but entire microorganisms’ cells are very promising because the microorganisms can easily be generated and used in the fermenters. The use of biotransformation systems allows biotechnology products to be labeled as natural. Market analysis indicates that customers prefer natural ingredients while artificial ingredients have many side effects like allergy, nausea, chest pain or headache and sometimes even detrimental consequences like cancer, negative effects of neurons, kidney damage, etc. (Roman et al., 2017).

Besides studying the chemical properties of natural volatile flavour compounds (VFCs), which cause aroma and flavours perceived, some studies have shown that their antioxidant, anti-cancer, anti-inflammatory, and anti-obesity activities may have potential applications to human health (Caron et al., 2021, Paulino et al., 2021). In addition, market demand shows a trend to natural goods, with the bio-generation of trade-relevant natural volatile aroma compounds, especially the synthesis or biological transformation by enzymes or whole cells in traditional aqueous solution, mainly centered within industry and academic sectors (Ayseli and Ayseli, 2016, Caron et al., 2021).

There is a great deal of curiosity about natural products. This drives the fragrance industry to create new methods for extracting compounds with natural aromas. Bioconversion is another form of this natural synthesis. It is well known that the production of volatile aroma compounds by enzymes or microorganisms for the food industry provides various advantages over conventional methods (Paulino et al., 2021). The use of solid-state fermentation in conjunction with submerged fermentation often provides higher yields or superior product features with reduced costs. Furthermore, owing to its high boiling point and high temperature at which it evaporates, water impedes the isolation and purification processes as well as the process integration (Try et al., 2018).

In food production, microbiological contamination can pose a health hazard by inducing diseases such as diarrhea, stomach cramps, vomiting, and even death. The intake of fresh and minimal processed foods has been seen a drastic rise in outbreaks of foodborne conditions in recent decades due to *Enterobacter aerogenes*, *Escherichia coli* O157:H7, *Listeria monocytogenes*, *Pseudomonas aeruginosa*, *Staphylococcus aureus,* and *Salmonella* (Al-fekaiki et al., 2017). The *in vitro* studies report the antimicrobial action of natural VFCs. The natural flavours of phenolic compounds enter the cell membrane and disrupt the lipid structure of the membrane. Theoretical abilities also disrupt the permeability of the membrane and impacts disrupt cellular ion gradients (Tometri et al., 2020).

**Fig. 1:**
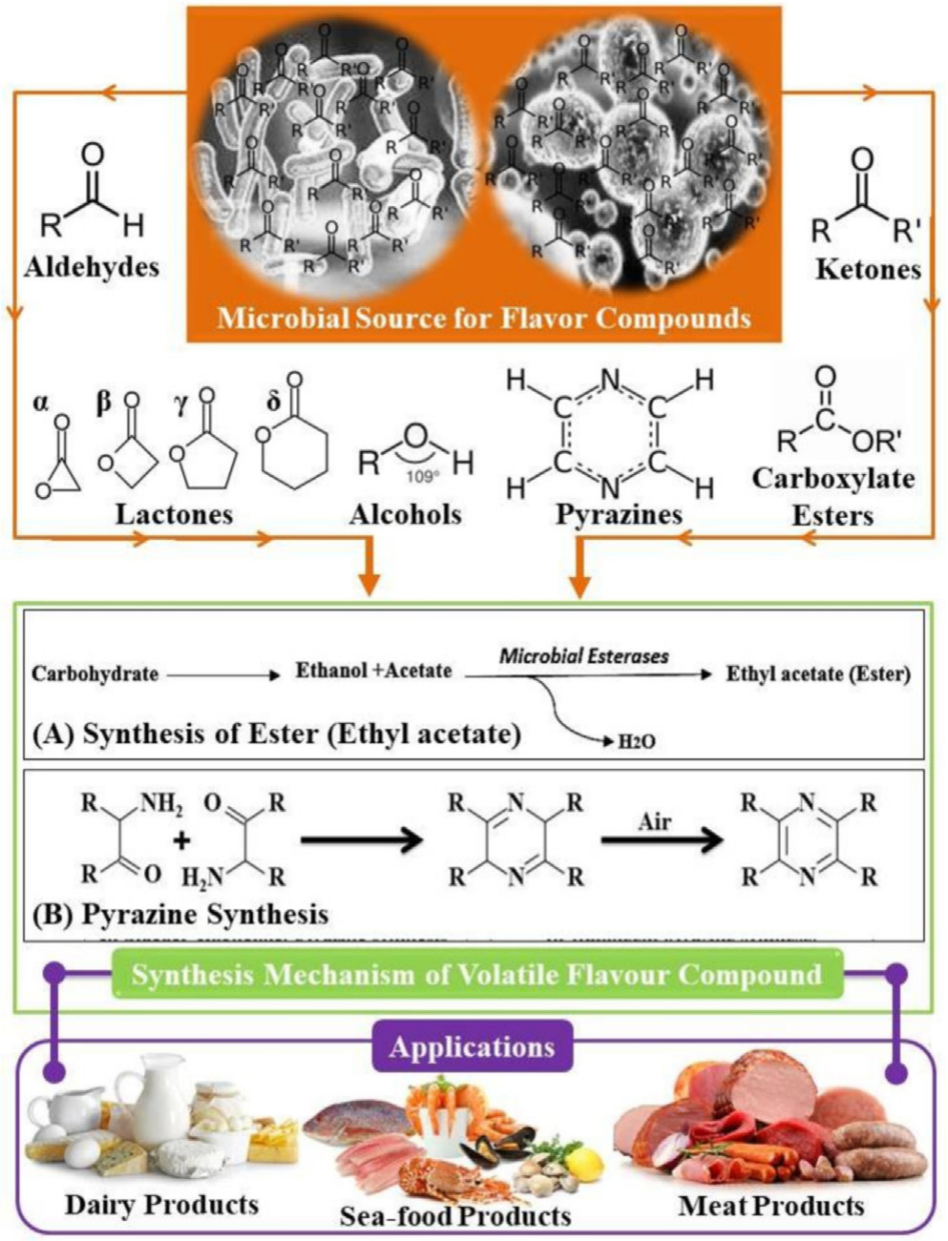
A graphical summary of this study of recent trends in microbial flavour compounds, chemistry, synthesis mechanism and their application in food.

In view of the above discussion, this study presents the current progress in several VFCs, their microbial synthesis as well as the high potential for commercial use in foods. The current review also assesses the antimicrobial potential of different flavour compounds from the enzymatic and microbial origin in addition to exploring their utilization in milk-based products, meat, and seafood processing. Fig. 1 accurately depicts the overview of this review study.

## 2 Types of microbial flavours compounds

**Fig. 2:**
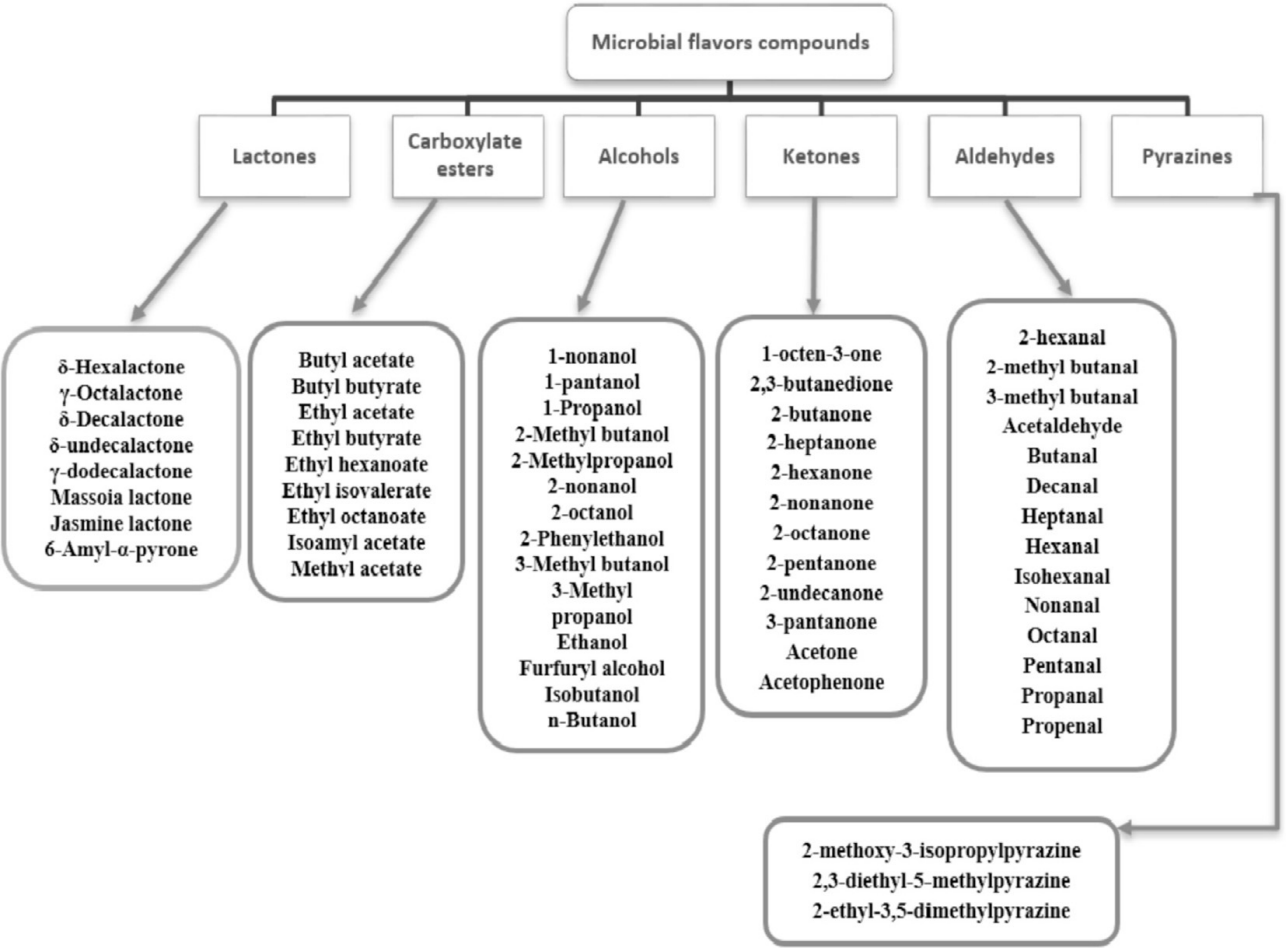
Classification on the basis of chemical compositions for microbial VFCs.

**Fig. 3:**
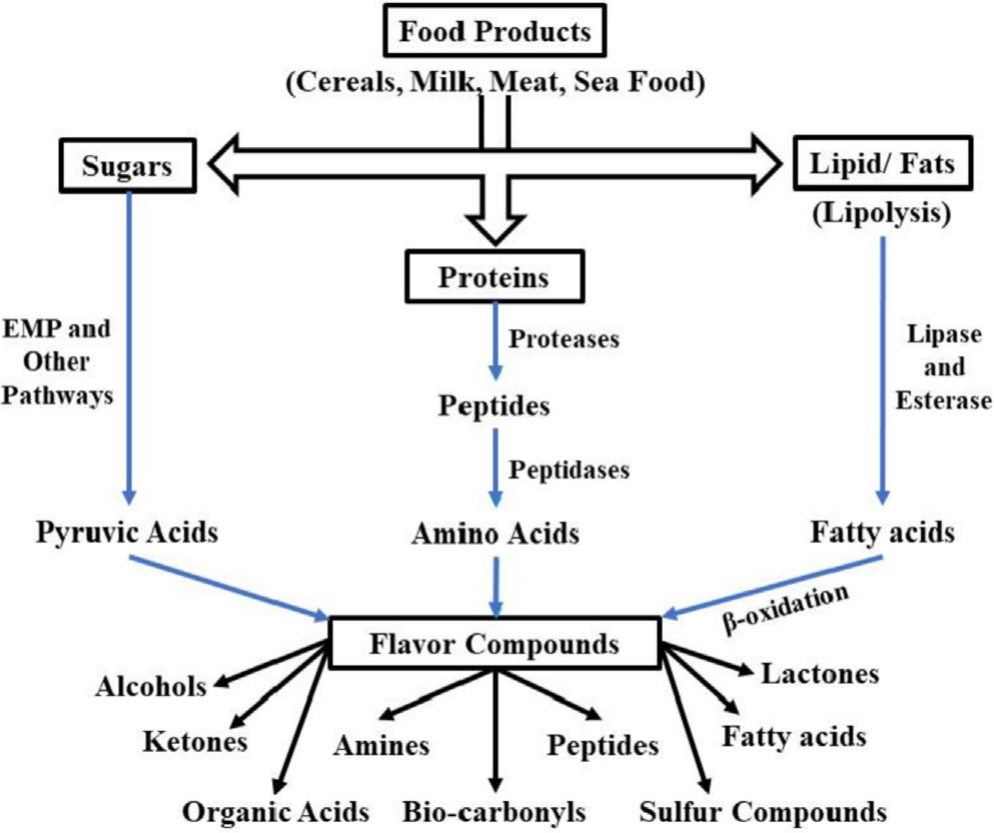
Biosynthesis of flavours by different microorganism in various food products.

Fig. 2 indicates the grouping of VFCs by chemical composition. Microorganisms have since already been used in many foodstuffs to produce flavours. Microbes are used to preserve, modify, and flavour products such as wine, beer, fermented vegetables and milk, soybeans, pickles, and meat vinegar. Different microorganisms follow the specific metabolic pathway to produce specific flavour compounds as summarized in Fig. 3. As mentioned above, microbial strains can either be used to produce VFCs *in-situ* or in suitable substrates from which VFCs are obtained and then used in various foodstuffs (Dan et al., 2019, Wang et al., 2020a, Wang et al., 2020b, Zinjanab et al., 2021). In Table 1**,** the outcomes of various researchers for the biosynthesis of VFCs from different substrates by specific microbial species are compiled. The following sections discuss the most relevant category of VFCs used in the food industry and their microbial production:

**Table 1:** Biosynthesis of flavours from different substrates by microorganisms.

**List of TablesCompound**	**Microorganisms Involved**	**Substrate**	**References**
δ-Decalactone	*Aspergillus niger*, *Cladosporium suaveolens* and *Pichia etchelisii*	Oil of *R. communis*	Prabakaran et al. (2020)
	*Monilia fructicola*, *Rhodotorula glutinis* and *Sporobolomyces odorus*	Castor oil	Bhari & Singh (2019)
	Yeast species	11-hydroxy palmitic in sweet potatoes (*Ipomoea batatas*)	Vandamme & Soetaert (2002)
Octalactones	*Mortinella* spp.	Octanoic acid (caprylic acid) found in coconut oil	Vandamme & Soetaert (2002)
Ethylacetate	Yeasts including *Cyberlindnera jadinii*, *Kluyveromyces marxianus,* and *Wickerhamomyces anomalus*	Sugars or ethanol	Fan et al., 2019, Zhang et al., 2020b
2-phenylethanol	*Saccharomyces cerevisiae*	Fermented wine	Liang et al.(2020)
2-phenylethanin	*Hansenula anomala*, *K. marxianus*, and *S. cerevisiae*	2-phenylalanine	Chantasuban (2016)
Diacetyl	LAB such as *Leuconostoc* sp*., Lc. lactis* subsp.*lactis* serover *diacetylactis*and Yeasts	Milk, Synthestic media	Rosca et al., 2016, Wang et al., 2019b
Acetaldehyde	Lactobacilli and Yeasts	Milk, Synthestic media	Dan et al., 2019, Rosca et al., 2016
Benzaldehyde	*Pseudomonas taiwanensis*(engineered)	Supplimented medium of glucose or glycerol	Otto et al. (2020)
Pyrazine	*Bacillus subtilis*	Fermented soyabean	Zhang et al. (2020a)

### 2.1 Lactones

Lactones are internally formed cyclic esters of γ- and δ-hydroxy acids which are in combination with their corresponding alcohols. These lactones have a wide flavour composition that contributes primarily to too many flavours in dairy products, including buttery, coconut, creamy, fruity, nutty, or sweet flavours. Free lactones and their precursors come from fresh butter with a sweet fruity fragrance. In animal fats as well as auto-oxidized vegetable oils, δ-lactones are also found. This gives candies and pastries a strong flavour whereas, the weak flavour of tea, strawberry, raspberry, coconut, and butter is due to the presence of 5-methylpentanolide or δ-Hexalactone (Bhari and Singh, 2019, Kendirci et al., 2020). Table 2 discusses some of the other major VFCs of the lactones group with their chemical structure and food sources.

**Table 2:** Food sources and chemical structure of some Lactones types.

Lactones Types	Chemical Structure	Food Sources
δ-Hexalactone		Fruit and milk products
γ-Octalactone		Yoghurt, peaches, oranges and sweet fortified wines
δ-Decalactone		Tea, blue cheese, tobacco, mango, strawberry and butter
δ-Undecalactone		Milk products
γ-Dodecalactone		Milk products and coconut
Massoia lactone		Tobacco, wine and molasses
Jasmine lactone		Jasmine and gardenia flowers, tobacco and tea
6-Amyl-α-pyrone		Animal foods, peach and heated beef

δ-Decalactone is a key lactone for the flavouring industry also called decan-4-olide and is used as a dairy and fruit flavour. It has an incredibly strong smell and a creamy taste with a concentration of<5 parts per million (ppm). Peach fruit is the best source of this VFC which can be used as the aroma of apricot (*Prunus armeniaca*), peach (*Prunus persica*), coconut (*Cocos nucifera*), date (*Phoenix dactylifera*), maple (*Acer* spp.), pear (*Pyrus* spp.) and butterscotch. This VFC can be commercially synthesized in a biochemical reaction that is catalyzed by *Candida guillermondii* lipase or *Yarrowia lipolytica* by the transformation of the ricinoleic acid found in the oil of the castor plant (*Ricinus communis*). δ-Decalactone from the oil of *R. communis* has been reported to be produced by the microorganisms such as *Aspergillus niger*, *Cladosporium suaveolens,* and *Pichia etchelisii* (Prabakaran et al., 2020). Some microbial species such as *Monilia fructicola*, *Rhodotorula glutinis,* and *Sporobolomyces odorus* have been used to report comparatively lower product yields (Bhari and Singh, 2019). The oleaginous yeast (*Yarrowia lipolytica*) engineered recently by **Marella and co-workers,** in which it has been used by beta(β)-oxidation to hydroxylate fatty acids (FAs) and to shorten-chain preferentially 12 or 10 carbons (Marella et al., 2020). The engineered strains have shown that γ-Dodecalactone and δ-Decalactone from oleic and linoleic acid respectively yield fourfold higher levels than the wild strain, thereby paving the way for higher lactone production by fermenting available fatty feedstocks. The dairy products are particularly attractive for the buttery coconut, and milky flavours of these microbiologically-based lactones (Marella et al., 2020).

The fragrance of coconut (*C. nucifera*) is due to 6-pentyl-2-pyron (6-PP) which has been reported to be a key VFC in the cultures of *Trichoderma viride*. The γ-dodecalactone and δ-Decalactone have been converted from coriolic acid and ricinoleic acid (Siddiquee, 2017, Khan et al., 2020). *N*-octanoic acid (caprylic acid) is found in coconut oil which has been fermented by *Mortinella* spp. to produce octalactones. Whereas, 11-hydroxy palmitic is found in sweet potatoes (*Ipomoea batatas*) which has also been reported to produce δ-Decalactone by fermentation of yeast species (Vandamme and Soetaert, 2002).

Previously, De Aráujo et al.(2002) evaluated the production of an unsaturated lactone 6-pentyl-a-pyrone (6-PP) with a strong coconut-like aroma, using both liquid and solid substrates in solid state fermentation (SSF) process where they used sugarcane bagasse as a substrate for growth and aroma production. In another approach, using mixed cultures of Lactobacillus acidophilus and Pediococcus pentosaceus, semisolid maize-based medium was utilized to produce flavour compounds like diacetyl, butyric acid and lactic acid by Escamilla-Hurtado et al. (2005).

### 2.2 Carboxylate esters

**Fig. 4:**
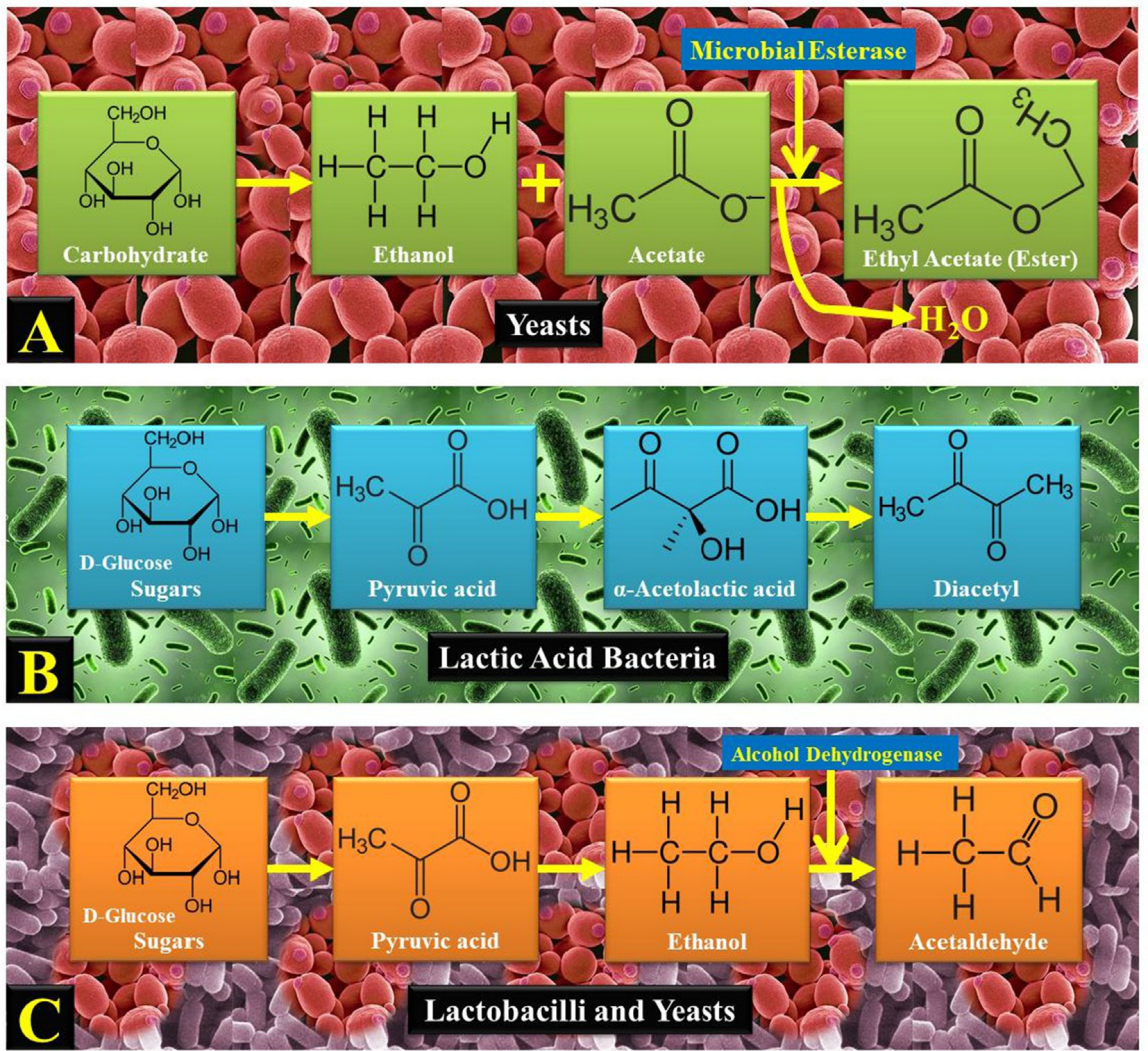
Synthesis of different microbial VFCs. (A) Ester (ethyl acetate) synthesis from carbohydrate through yeasts, (B) Diacetyl synthesis from carbohydrate in LAB, (C) Acetaldehyde synthesis from carbohydrate in lactobacilli and yeasts.

Natural microorganisms for ester synthesis are well known and have traditionally been used in food production, such as lactic acid bacteria (LAB) and yeast. Of these esters, volatile esters are the most important aromas in fermented foods, including beer, dairy, and wine products. Ester produces a pleasant, fruity fragrance at low concentrations, but is also considered to be off-tasting when found in significant amounts. Ethylacetate is the highest volatile ester in food. The concentration of such volatile ester in dairy products varies between ∼ 50 and 100 mg/L (Wang et al., 2016) and in beer and wine between ∼ 0 and 60 mg/L (Daniel et al., 2015). There are several more volatile esters, including ethyl hexanoate, isoamyl acetate, and phenylacetate, but concentrations of more than 1 mg/L have not been observed (Dzialo et al., 2017). These amounts lying at or just above the threshold for identification by human beings impair the scent of food items significantly (Dzialo et al., 2017). While there are some exceptions, including the mass production of acetate-generated yeast or wax ester production by *Euglena gracilis* (Inui et al., 2017), the amount of natural ester from microorganisms is usually small. Under anaerobic conditions, wax esters have been documented to accumulate as much as 65% of the dry cell weight (Inui et al., 2017). Ethylacetate from sugars or ethanol can be synthesized by yeasts including *Cyberlindnera jadinii*, *Kluyveromyces marxianus,* and *Wickerhamomyces anomalus* (Fig. 4 **A**) (Fan et al., 2019, Zhang et al., 2020b). There are also other enzyme groups that can form ester but for ester synthesis, they have not been used widely (Xu et al., 2020).

### 2.3 Alcohols

Unsaturated alcohols have been used in food ingredients with a distinctive scent. Many types of yeast contain complex alcohols with a long chain of organoleptic properties (Wang et al., 2019a). The batch of fermented wine with *Saccharomyces cerevisiae* was immobilized by **Liang and coworkers** with a fine cellulose material, who produced a rose-like scent, 2-phenyl ethanol, in a fermented wine (Liang et al., 2020). This offers an alternative microbial path for the synthesis of alcohol, which is usually isolated from rose petals or chemically synthesized. *Hansenula anomala*, *K. marxianus*, and *S. cerevisiae* have been identified for the production of 2-phenylethanin from 2-phenylalanine (Chantasuban, 2016). Noteworthy, *K. marxianus* was engineered by **Kim and coworkers** to overexpress genes that encode enzymes (such as alcohol dehydrogenase and phenylpyruvates decarboxylase) from *S. cerevisiae* that contribute to the overproduction of 2-phenylethanol from glucose (Kim et al., 2014). *S. cerevisiae* is an alternative ethanol synthesis microorganism to *Zymomonas mobilis*. The growth and synthesis of ethanol from four isolates were contrasted with the growth and synthesis of the efficient strain *Z. mobilis* (NRRL B-14023) at different temperatures in order to detect the thermotolerant strain of *Z. mobilis* (Xia et al., 2019). In *Z. mobilis*, glyceraldehyde-3-phosphate-to-pyruvate, and pyruvate-to-ethanol pathways, the ethanol production route of glucose, consisting of the Entner-Doudoroff path, provides the majority of ATP needed for cell activity for approximately one mole of ATP per mole of glucose (Xia et al., 2019).

### 2.4 Ketones

Ketones are carbonyl molecules (=C = O) that trigger a lot of natural flavours and smell. Several saturated as well as unsaturated aromatic, aliphatic, and cyclic ketones have been reported from the cheese (Caron et al., 2021, Ianni et al., 2020). Among them, unusually C_5_-C_11_ numeric ketones, such as 2-alkanones along with secondary alcohols and free fatty acids (FFAs), which may have their distinctive aromas in *Penicillium*-ripened cheese (Caron et al., 2021). Diacetyl is known as a butter-flavoured vicinal diketone, and is therefore used for the simulation of butter-like and other milky flavours (Wang et al., 2019b). It is produced in dairy foods by various microorganisms including lactic acid bacteria (LAB) (Wang et al., 2016, Wang et al., 2019b). In Fig. 4 **B**, the biosynthesis of diacetyl in LAB from carbohydrates is depicted. It is interesting to note that the metabolically engineered *Enterobacter clocae* provided high levels of diacetyl (1.45 g/L), while diacetyl and acetaldehyde were produced by yeasts *Candida tropicalis* strain D15 in the whey-based medium (Rosca et al., 2016). The essential components of the cooked flavour of the butter-containing baked food are methyl ketones. Methyl ketone precursors occur as alkanoic acids in fresh butter with no aroma features. However, they are converted into methyl ketones, which are the main VFCs in cooked and heated foods containing butter. These VFCs are formed by *A. oryzae*, *A. niger*, *A. bisporus*, *Penicillium roquefortii,* and *T. viride*. The mechanism of β-oxidation of FAs can be used to rapidly produce methyl ketones in microbes (Sharma et al., 2020a, Sharma et al., 2020b).

### 2.5 Aldehydes

Vanillin is a 4-hydroxy-3-methoxybenzaladehyde and is considered a very effective VFC used in food for industrial purposes. The extraction of this VFC vanillin from vanilla pods is difficult due to labor-intensive and costly production. Due to increased consumer demand for natural vanillin, there is no need for chemically synthesized vanillin. As a result, several groups of researchers have obtained vanillin from essential oils by the microbial conversion of eugenol and isoeugenol (Ashengroph and Amini, 2017). Surprisingly, lignocellulosic obtained from agricultural residues have been discussed as a rich source of ferulic acid in a recently published report by Sharma and coworkers (Sharma et al., 2020a, Sharma et al., 2020b). Furthermore, the use of ferulic acid has also been addressed in vanillin synthesis through microbial or enzyme transformations (Sharma et al., 2020a, Sharma et al., 2020b). However, this procedure requires the synthesis of vanillin in which ferulic acid is released from lignocellulosic waste by enzyme or chemical intervention. Some bacteria and fungi may transform the released ferulic acid into vanilla, vanilla, and protocatechuic acid. The role of ferulic acid in the field of bioflavour production has therefore been recognized as a precursor to vanillin synthesis (Tang and Hassan, 2020, Sharma et al., 2020a, Sharma et al., 2020b). Vanillin has been biotransformed into ferulic acid by numerous microbial species (Tang and Hassan, 2020), including *Actinomycetes* spp*., Amycolatopsis* spp., *Aspergillus* spp. (*A. niger*), *Bacillus* spp. (*B. coagulans*, *B. licheniformis,* and *B. subtilis*), *Corynebacterium* spp. (*C. glutamicum*), *Debaryomyces* spp. (*D. hanseni*), *Escherichia* spp. (*E. coli*), *Halomonas* spp. (*H. elongata*), *Pseudomonas* spp. (*P. fluorescens* and *P. putida*), *Pycnoporus* spp. (*P. cinnabarinus*), *Rhodococcus* spp., *Rhodotorula* spp. (*R. rubra*), *Saccharomyces* spp. (*S. cerevisiae*), *Schizophyllum* spp. (*S. commune*), and *Streptomyces* spp. (*S. halstedii*, *S. sannanensis*, and *S. setonii*). For the synthesis of cherry and fruity flavour, benzaldehyde is the second largest aldehyde next to the vanillin. It can be obtained from *P. armeniaca* (apricots), but the procedure results in unwanted hydroxycinnamic acid accumulation. Additionally, microbial synthesis of benzaldehyde from phenylalanine may be considered as “natural” without undesirable by-products. In this context, the engineered *Pseudomonas taiwanensis* strain has been found to synthesized benzaldehyde in supplemented medium of glucose or glycerol (Otto et al., 2020)*.* In fermented milk like yoghurt, acetaldehyde is the key flavouring compound formed by several *Lactobacillus* sp., further few types of yeast were also found to synthesize acetaldehyde (Dan et al., 2019, Rosca et al., 2016**).** In Fig. 4 **C**, the biosynthesis of acetaldehyde from carbohydrates is depicted.

### 2.6 Pyrazines

**Fig. 5:**
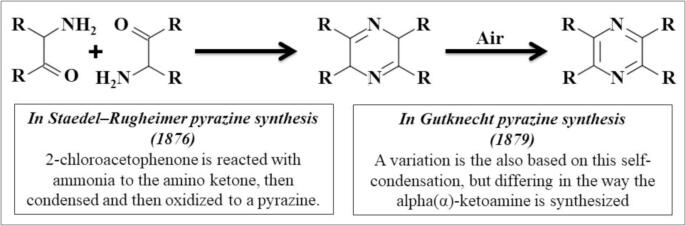
Pyrazine synthesis by condensation reaction.

Pyrazines are heterocyclic molecules that contain nitrogen and produce nutty and roast flavours (Mortzfeld et al., 2020). The main factors for green flavours in sauvignon Blanc wines are 3-isopropyl-2-methoxy pyrazine and 3-isobutyl-2-methoxy pyrazine. Furthermore, there are also several other essential chemical compounds which have been considered as flavour enhancers. For example, 2,3,5-trimethyl pyrazine, 2-acetyl-3-methoxy pyrazine, 2-methyl-3-isobutyl pyrazine, and 2-methyl-3-methoxy pyrazine improve the flavour of chocolate, almonds, bell pepper, and toasted corn (Mortzfeld et al., 2020, Guo et al., 2021). There are many available approaches used for the organic synthesis of pyrazine and its derivatives. Some of the proposed approaches are the oldest, and some are recent. The reactions used for synthesis in the oldest methods are Staedel–Rugheimer pyrazine synthesis (1876) and Gutknecht pyrazine synthesis (1879) still in use, with variations in each other (Fig. 5). Historically, the first microorganism reported for pyrazine synthesis was *Bacillus subtilis* (Zhang et al., 2020a). Previously, tetramethylpyrazine (TMP) and 2,5-dimethylpyrazine (2,5-DMP) were synthesized from acetoin, D-glucose, and L-threonine using the strain *B. subtilis* 168 (Zhang et al., 2019). In addition, pyrazines synthesis from amino acids (AAs) has also been reported using *Corynebacterium glutamicum* (Eng et al., 2020). Nutty and chocolate flavouring chemical pyrazines have recently been synthesized by Fadel and coworkers using *C. glutamicum* grown on soyabean (*Glycine* max) with enriched lysine and threonine medium (Fadel et al., 2018). Maillard reactions can also produce pyrazines in conventional cooking and roasting. However, pyrazines have not been synthesized due to advances in cooking techniques, such as the use of microwave ovens. We can therefore conclude that roast flavour as food additives must be provided with natural pyrazines.

LABs are able to synthesize different flavours by catabolism amino acids. Initially, the amino acids are involved in dehydrogenation and transamination reactions and forms α-ketoacids, a compound which have a primary effect on flavour amount and type. Moreover, α-ketoacids are converted in aldehydes through decarboxylation reaction (Petrovici and Ciolacu, 2018). In one of the researches, development of phenylacetaldehyde (with honey-like aroma) was identified by *Lb. helveticus, Lb. plantarum* UC1001, and *S. thermophilus* from catabolism of methionine (Siragusa et al., 2011). The same strains also showed synthesis of propionic acid from catabolism of threonine. Similarly, the biosynthesis of the diacetyl by some Lactobacillus strains from aspartate has been reported by Garde et al. (2007) while Zuljan et al. (2016) showed the formation of α-keto-β-methyl valerate from isoleucine catabolism.

## 3 Antimicrobial activities and action mechanism of flavour compounds

All the antimicrobial compounds have not yet been studied for their specific mechanisms for action. Unlikely 2-(*E*)hexenal appears to behave as a surfactant but probably penetrates across the plasma membrane with passive diffusion. The alpha(α)- and beta(β)-an unsaturated movement of aldehyde while within cells react with biologically significant nucleophilic classes. This aldehyde movement has mostly reacted by 1.4 additions in physiological conditions with sulfhydryl groups (Hayashi et al., 2019). Sulfhydryl groups are considered to play an important function in living cells in protein, and lower-molecular compounds such as glutathione (Li et al., 2020b). The mode of action of the bioactive compounds of spices, herbs, and plants are more complex to assume since they also have varying quantitative and qualitative ratios of essential oils. They may vary greatly from the target microorganisms and the minimum inhibitor concentrations in their microbicidal and microstatic effects (Merveille et al., 2017). The techniques of analyzing antimicrobial activities and, in particular, the protocols followed for the extraction, diffusion, and dilution of chemical compounds may be due to certain inconsistencies in the bioactivity assessment of those chemical compounds. Essential oils are combinations of chemicals compounds that also have low water solubility and high hydrophobicity (Sarıcaoglu and Turhan, 2020). In the previous study, **Janssens and coworkers** evaluated *Paenibacillus* sp. AD87 which demonstrated antifungal activity because of the 2,5-*bis*(1-methylethyl)-pyrazine during a co-culture with *Burkholderia* sp. AD24. In addition, *E. coli* and mammalian cells were used to decipher a potential mode of action with transcriptional reporter tests. Strains of mammalian and bacterial luciferase reporter were also used to elucidate 2,5-*bis*(1-methylethyl)-pyrazine antimicrobial and toxicological effects. 2,5-*bis*(1-methylethyl)-pyrazine had a good reaction to DNA damage at elevated exposure levels (Janssens et al., 2019). During a 15 days storage time, at 4 ± 1 °C, the mixture of vanillin and chitosan coating had an influence upon the microbiota composition and duration of turbot filets (*Scophthalmus maximus*). The relative abundance of the *Lactobacillaceae* and *Pseudomonadaceae* had decreased significantly following vanillin and chitosan treatment due to the growth inhibition of possible bacteria, particularly spoilage bacteria, and the rich end of storage body diversity (Li et al., 2020a). Moreover, *Photorhabdus temperata* produced benzaldehyde showed a strong antioxidant activity (AOA) and a maximum AOA at 8 mM compared to a control of 52.9 %. MIC values of 6 mM to 10 mM for bacterial strains and 8 mM to 10 mM for fungal strains were measured for antimicrobial activity (Ullah et al., 2015).

Strains of LAB and bifidobacteria could produce diacetyl in up to 30 mg/mL concentrations and had a potential to exhibit antimicrobial activities, especially against Gram-negative bacteria (such as i, *Pseudomonas aeruginosa*, *Salmonella typhi, Pasteurella multocida*, *Klebsiella rhinoscleromatis,* and *Bartonella* sp.*,*) as well as against fungi (Lew and Liong, 2013, Patel and Shah, 2014). Another direction of using diacetyl is related to active packaging systems where the controlled release of volatile antimicrobial compounds is possible through packaging material. In this context, in conjunction with 20% CO_2,_ effects of diacetyl were evaluated on the quality of ground beef while using it in modified-atmosphere packaging. It was associated with the fresh colour and odour of the products as well as a delayed spoilage of product (Williams-Campbell and Jay, 2002).

## 4 Microbial flavours compounds in food

Fermentation is a common way of preserving and preparing foods. In different foods, it can produce specific aromas and flavours. Microbial flavours are one of the food's most significant qualities and are closely linked to consumer product approval. In the past decades, the interest in the biotechnological production of VFCs has increased in various ways to achieve these compounds as this technique has been seen as a sustainable means of providing the food industry with natural additives (Dan et al., 2019, Paulino et al., 2021). Due to their mild environments, their biotechnological synthesis of VFCs is not subject to possible toxic catalysts and the problems of waste treatment are considered an ecologically safe solution. Furthermore, agro-residues can be used as a substitute, ecologically as well as economically beneficial raw materials for this bioprocess. The waste of cellulose, lignocellulose, and starch can also be used for the synthesis of VFCs like aldehydes, alcohols, ketones, FAs, esters, terpenes, pyrazines, and lactones. Furthermore, industrial fermentation does not require extractive considerations for the processing of flavours (Try et al., 2018, Sharma et al., 2020a, Sharma et al., 2020b). Different volatile compounds have been detected through gas chromatography and other combined treatments based on the type of fermenting microorganisms and food products (Table 3**).**

**Table 3:** Presence of volatile compounds in different food products by gas chromatograpgy.

**Type of Food Product/Sample**	**Details of GC Column**	**Major Finding(s)**	**Reference**
Suan-zuo-yu	7890B gas chromatography coupled to 5977B mass selective detector with a VF-WAXms capillary column (30 m length × 141 0.25 mm inner diameter × 0.25 μm film thickness)	GC–MS showed a complete identification of 80 VFCs and a significant increase in aldehydes, alcohols and esters, which mainly led to the flavour of the product by LAB fermentation.	Wang et al. (2020b)
Ewes’ milk cheese	DB5 capillary column, 0.32 μm internal diameter, 1 μm film thickness, 60 m long	The levels of several volatile organic compounds were significantly (P < 0.05) lower in control cheese than in cell-free extracts-supplemented cheeses. All cheeses manufactured by adding multiple CFEs exhibited higher scores (P < 0.05) for internal structure, juiciness, and acid taste than control samples.	Calasso et al. (2017)
Sliced cooked pork	ZB-WAXplus polyethylene glycol capillary column, 0.25 mm internal diameter; 0.50 μm film thickness, 60 m long	At the end of the storage period, 500 and 600 MPa samples contained higher levels of branched-chain aldehydes, ethanol, diacetyl, acetoin, and 2,3-butanediol whereas control and 400 MPa samples showed higher levels of fatty acids and ethanol and ethyl acetate esters.	Rivas-Cañedo et al. (2011)
Pecorino Abruzzese cheese	CP-Wax 52 CB polyethylene glycol coated, 0.32 mm, 1.2 mm film thickness, 50 m long	The analyses of volatile compounds revealed the production of diacetyl, ethanol, and acetoin after 15 days at 10 °C, with important differences among the Enterococcus sp.	Serio et al. (2010)
Morcilla de Burgos	HP-5MS capillary column, 5% phenyl methyl silicone, 320 μm, 1.0 μm, 60 m long	W. viridescens samples showed greater amounts of alcohols (ethanol) and ketones (acetoin and diacetyl) whereas L. mesenteroides samples were richer in aldehydes (hexanal) and acids (acetic).	Diez et al. (2009)
Cheese	HP-INNO-WAX polyethylene glycol capillary column, 250 μm, 0.5 μm, 60 m long	The mixture of L. lactis IFPL326 led to the highest formation of leucine-derived volatile compounds like 3-methylbutanal, 3-methyl-1-butanol and 2-hydroxy-4-methyl pentanoic acid methyl ester through aminotransferase activity with IFPL730	Amárita et al. (2006)

The following section discusses the VFCs of enzymatic and microbial origin, as well as the progress in their potential and commercial use in food.

### 4.1 In dairy products

Traditional fermented foodstuffs and drinks are relatively like complex microbial habitats which can be used as microbial models to explain the interaction of microbes in natural ecosystems because of the diverse essence and patterns of the kefir fermentations (Misihairabgwi and Cheikhyoussef, 2017). The previous researches have revealed the relationship between different microorganisms and their associated pathways for synthesis of VFCs, and established multiple genes responsible for the alleged wellness related to the protection of the gut used by kefir (Walsh et al., 2016, Misihairabgwi and Cheikhyoussef, 2017). This knowledge can ultimately be used to refine the fermentation mechanisms, flavours, and health properties of this and other fermented foods, in addition to providing an important fundamental insight into microbial interactions. In kefir milks formed from each of the three kefir grains, thirty-nine volatile aroma compounds were detected and semi-quantified. This included aldehydes (7), ketones (9), esters (6), alcohols (8), carboxylic acids (5), and sulfur-containing (2) compounds. In addition to acetone, butanone, heptanal, heptanol, hexanal, 1-pentanol, and pentanal, the amounts of all reported VFCs rose during storage time (Walsh et al., 2016).

The main routes of VFCs synthesis in cheese are due to lactate and lactose metabolism. The lactate may be transformed into different compounds that lead towards the flavour of the cheese in the first path, which completely depends on the variety of cheese, employed microflora, and conditions of ripening (Ianni et al., 2020). The other route produces fat-derived chemical compounds, such as esters, FFAs, ketones, and lactones, produced by lipolysis, lipid oxidation reactions with low aroma levels (Thierry et al. 2017). The aroma of cheese is due to VFCs produced by the action of the enzyme during the ripening.

Cheese from the Uyghur Autonomous Region of China, a characteristic handmade fermented milk food is known as Kazak cheese (Zheng et al., 2021). Recently, Zheng and coworkers investigated bacterial microbiota and VFCs during the milk fermentation of Kazak cheese. Headspace solid-phase microextraction (Hs-SPME) and Illumina MiSeq sequencing technologies were used in their investigation as analytical instrumentation coupled with gas chromatography/mass spectrometry (GC–MS). Dominant populations such as *Lactococcus* and *Lactobacillus* were reported during the fermentation of the milk. The relation between the flavour dynamics and the succession of the microbiome was defined based on the bi-directional orthogonal partial least squares (O_2_PLS) in which eight genera of bacteria were identified as the main functional microbes for the synthesis of flavour (Zheng et al., 2021).

. Since the flavour synthesis of natural milk fat, interest in the application of biocatalysts has gradually increased. Lipases are a class of enzyme which have been most studied as biocatalysts for the synthesis of flavour from bovine milk fat (Omar et al., 2016, Kendirci et al., 2020). In earlier studies of Omar and coworkers, anhydrous milk fat (AMF) and anhydrous buffalo milk fat (ABMF) were hydrolyzed using *Thermomyces lanuginosus* immobilized (TL-IM) lipase, Lipozyme-435, and Novozyme-435 (Omar et al., 2016). In addition, SPME and GC–MS were used to study the VFCs of AMF and ABMF. Omar et al. (2016) compared these VFCs at three intervals of hydrolysis. After the lipolysis of AMF and ABMF, the Novozyme-435 and Lipozyme-435 produced the highest hexanoic and butanoic acids as well as other VFCs, followed by TL-IM. Rancimat-743 evaluated hydrolyzed materials for oxidative stability, both of which showed that butter oil-treated for AMF and ABMF was relatively more stable in Lipozyme-435 and TL-IM compared with Novozyme-435. Lipozyme-435 was found not to induce additional oxidation effects indicating that Lipozyme-435 was stable during 24 h, at 55 °C, for both AMF and ABMF butter oil-treated (Omar et al., 2016).

Calasso et al. (2017) carried out measurement of volatile compounds produced in Ewes’ milk cheese through gas chromatography. Authors stated that the levels of several volatile organic compounds were significantly (P < 0.05) lower in control cheese than in cell-free extracts (CFE)-supplemented cheeses. All cheeses manufactured by adding multiple CFEs exhibited higher scores (P < 0.05) for internal structure, juiciness, and acid taste than control samples. Similar to this, in a previous approach, in Pecorino Abruzzese cheese the analyses of volatile compounds observed the production of diacetyl, ethanol, and acetoin after 15 days during storage at 10 °C with important differences among the Enterococcus sp. (Serio et al., 2010**).**

### 4.2 In meat products

More than 200 different volatile compounds have been identified from fermented meat items (Sharma et al., 2020a, Sharma et al., 2020b). Changes in physicochemical, microbiological, and sensory properties are caused by storage. A range of studies has been conducted to address the effect of VFCs responsible for the production of ripened aroma (Sidira et al., 2016, Perea-Sanz et al., 2019, Perea-Sanz et al., 2020, Silva et al., 2020). Perea-Sanz et al. (2019) described short vacuum storage times and a modest nitrate reduction (15%) in fermented sausages linked to odor-producing compounds (2,3-pentanedione, 3-hydroxy-2-butanone, ethyl octanoate, and ethyl-3-methylbutanoate) and buttery/cheesy odor (ethyl-2-hydroxypropanoate and 2,3-butanedione). While in the freshness of cured pork loins, Silva et al. (2020) reported that the loss was mainly due to decreased aromatic note values (especially the smokiness and cure) and to the presence of spoiled characteristics, primarily the aroma of sauce/vinegar and acidic flavour identified. According to a reduced ratio of respondents, *Listeria monocytogenes* inoculated in the cured pork loins slices did not result in a reduction in the freshness of pork slices.

In addition, the effect of *Debaryomyces hansenii* inoculation on the production of fragrance was examined to reduce the ingoing levels of nitrite (NO^−2^) and nitrate (NO^−3^) in dry fermented sausages. Different drying periods have been examined for modifications in physicochemical and microbiological parameters, flavours, and VFCs (Perea-Sanz et al., 2020). The reduction in NO^−2^ and NO^−3^ did not seem to affect microbial development, but on their metabolic function. *D. hansenii* inoculation, which led to the generation of strong compounds such as ethyl ester and 3-methyl butanal, had a beneficial impact on the aroma profile of sausage (Perea-Sanz et al., 2020).

High counts of LAB were mildly affected by the diameter of dry fermented sausages of the Milan type, whereas higher Staphylococci concentrations were found in small sausages. The diameter had a significant effect on the production of VFCs like aldehydes (mainly hexanal) and ketones (acetone, 2,3-butandione, 2-butanone, and 3-hydroxy-2-butanone,), which showed the major variations. Even the appearance of the indigenous *Lactobacillus sakei* in the saucers inoculated with pediococci showed a less obvious influence on the starter cultures (Montanari et al., 2018). The starter culture has a direct impact on certain key process parameters (acidification and fermentation rate) and VFCs generation for all forms of fermented foods.

For the reason that aldehydes send out green grass, nutty, candy and cheese odor and have low threshold values, aldehydes are considered as essential compounds to the flavor development of fermentation meat (Dajanta et al., 2011).

### 4.3 In sea food products

One of the essential quality characteristics of fermented fish is its distinctive flavour. The studies on the formulation in flavours of fermented fish products have been focused primarily on isolation, purification, and characterization of VFCs and their biosynthesis process. The breakdown of protein substrates with the effect of enzymes obtained from both the microbes and fish have also been described to produce a specific flavour during the spontaneous fermentation of fish (Marti-Quijal et al., 2020). In a recent report, the fermentation was classified into two phases during the Mandarin fish fermentation based on microbiota changes: early, 1st-3rd, and late, 4th-7th, days. The typical VFCs of Mandarin fish fermentation were anethole, indole, linalool, piperitone, 2-methyl-3-octanone and 1-octen-3-ol (Yang et al., 2020). The large chain protein, myosin, and actin were decreased during the fermentation process of Suan-zuo-yu by LAB. GC–MS showed a complete identification of 80 VFCs and a significant increase in aldehydes (6), alcohols (6), and esters (6), which mainly led to the flavour of Suan-zuo-yu (Wang et al., 2020b).

Previously, GC–MS was also used for the analysis of VFCs (Wang et al., 2016**, 2017**). In 2017, GC–MS was used to identify acids, alcohols, aldehydes, esters, furans, hydrocarbons, ketones, and nitrogen-containing compounds (Wang et al., 2017). These were the main VFCs produced by the process of fermentation of fish. Wang and coworkers investigated fermented fish flavour and showed that *S. cerevisiae* 152 had degraded aromatic AAs (phenylalanine) and branched-chain AAs (leucine, isoleucine, and valine) to produce phenyl-ethanol, 2-methyl-butanol, 2-methyl-propanol, and 3-methyl-1-butanol. Furthermore, degradation of leucine and phenylalanine were also reported, resulting in the synthesis of phenylethanol and 3-methyl-1-butanol from *Lactobacillus pentococcus* 22 and *Lactobacillus plantarum* 120, respectively (Wang et al., 2017).

Esters are essential VFC in Suanyu fermentation that is indirectly implicated in FFA metabolism, contributing to the biosynthesis of esters due to esterification and alcoholic response. LAB was found to encourage acetate compounds production whereas *Staphylococcus* and yeast would promote ethyl compounds production (Sidira et al. 2016). As described above, microflora metabolic activities can produce a range of volatility, which can contribute eventually to the taste and consistency of metabolism of AAs and FAs. The addition of metabolic flexibility and microbial diversity may also provide the opportunity for innovative and enhanced products.

LABs are beneficial microorganisms for fermentation which could generate bioactive peptides and produce vitamins in fermented foods like fishes (**Şanlier et al., 2019;** Patel et al., 2013). In a study of Wang et al. (2020b), the levels of *Weissella* and *Lactobacillus* gradually increased from 0.311% to 46.00% suggesting that LAB play a vital role in fermentation of a traditional Chinese fermented fish product Suan-zuo-yu. Authors stated that increase in *Staphylococcus* was accompanied with the increase of esters while *Macrococcus* able to hydrolyze proteins and lipids was also presented throughout the fermentation process. In a previous study, *Macrococcus* was also detected in Chouguiyu, a kind of traditional Chinese fish product by Dai et al., 2013, Marui et al. 2014 found *Lactobacillus* and one *Weissella* sp. as a predominating bacteria from pa*-som*, a traditional fermented fish product in Laos.

Acetaldehyde plays a role in the rate of fermentation and the quality of wine. In a recent investigation, higher acetaldehyde levels were found in wines inoculated with *Saccharomyces cerevisiae*, exposed to high sulphur dioxide (SO_2_) levels, and fermented at higher temperatures (October 2020). There was a direct correlation between total ADH activity and total acetaldehyde production of *S. cerevisiae* yeasts.

## 5 Factors affecting production of microbial flavours

The compositions of growth media (including nitrogen and carbon source types), temperature, mineral composition, and level of aeration have a great influence on the biosynthesis of flavours by any microbial strain. For an instance, Paterson and Piggott (2006) stated that the addition of sucrose in the culture media stimulates the flavour biosynthesis for LABs and yeasts. With respect to this**,** Di Cagno and co-workers (2009) supplemented the tomato juice with sucrose and subsequently subjected to LAB fermentation in order to encourage the flavour production and also to lessen the intrinsic acidic flavour of tomatoes. Earlier, Cheetham (1999) in a milk or whey medium supplemented with citric acid as a precursor, a high diacetyl concentrations of up to 14 g/L have been stated for a patented process making use of *Streptococcus cremoris* and *S. diacetylactis*.

Similarly, the presence of oxygen strongly affect the growth of microbial strain and hence, the flavour biosynthesis. Under aeration conditions, *Lb. casei* leads to higher biosynthesis of diacetyl in Cheddar cheese than an anaerobic starter culture (Reale et al., 2016). On the other side, *E. faecium* FAIR-E 198 strain showed diacetyl biosynthesis only in aerobic conditions (De Vuyst et al., 2011).

The fermentation temperature strongly influences biosynthesis of flavour by microorganism. For example, *Lb. rhamnosus* ATCC 7469 biosynthesized diacetyl and acetoin within a temperature interval of 22–45 ˚C from citrate (De Figueroa et al.,) The maximum biosynthesis of diacetyl was observed in the temperature interval between 30 and 37 ˚C within 48 h; as compared to 22˚C, the level of the diacetyl and acetoin was 4.1 time higher at 37˚C. This effect is chiefly associated with the enzymatic activities in the microbial cell.

The presence of minerals also affects flavour production in microbial cells. In *Lb. plantarum* van Kranenburg et al. (2002) characterized two manganese transport systems which are implicated in mineral uptake and convert phenylalanine to benzaldehyde by initiation of a pyridoxal 50-phosphate-dependent aminotransferase. Further, in the presence of oxygen and manganese the obtained phenyl-pyruvate is further chemically transformed to benzaldehyde. In another research, it was observed that magnesium and manganese sulphate enhanced biomass and aroma development both of different 52 yeasts by obtaining 3.58 mg/L diacetyl for *Candida globosa* and 96.05 mg/L acetaldehyde for *Candida lipolytica* (Rosca et al., 2016).

In depth knowledge of the metabolism in microorganism has led to develop innovative strategies for engineering microbial strains with high flavour production in recent years. In a fibrous-bed bioreactor running under fed batch conditions immobilization of *Propionibacterium acidipropionici* ATCC 4875 led to 72 g L-1 propanoic acid (Suwannakham and Yang, 2005) while in separate investigation, knocking out the *ack* gene (acetate kinase) diminished unwanted acetic acid formation by 14% (Suwannakham et al., 2006).

## 6 Concluding remarks

The biotechnological synthesis of VFCs has gained popularity because of market demands for natural products and enhanced economic benefits. Low-cost natural precursors can be converted by microbes and their enzymes into costly VFCs and microbial synthesis methods benefit against conventional methodologies. Bacterial metabolisms can be used to produce different biocatalytic instruments to produce natural and fragrant value-added compounds and chemical synthesis from inexpensive plant biomass. Future directions to produce natural VFCs should be opened up by biotechnological advances. However, there should be solved certain concerns such as parent compound's toxicity, product toxicity, targeted gene expression and recombinant bacterial strain's physiological stability, in order to obtain costly “natural” flavours from genetically modified microorganisms (GMOs). Furthermore, the challenges faced by GMOs should also be addressed.

## Contribution of the authors

AKN, DKV, and STGA have conceptualized, interpreted, corrected, and made scientifically sound final versions of the manuscript; while, MT, ARP, NS, SS, DB, GLU, and CAN have provided technical suggestions and corrections for the final version of the manuscript. Funding acquisition has been provided by CAN. All authors critically reviewed and approved the final version of the manuscript for submission.

## Declaration of Competing Interest

The authors declare that they have no known competing financial interests or personal relationships that could have appeared to influence the work reported in this paper.
